# Damage of brown planthopper (BPH) *Nilaparvata lugens* and rice leaf folder (LF) *Cnaphalocrocis medinalis* in parent plants lead to distinct resistance in ratoon rice

**DOI:** 10.1080/15592324.2022.2096790

**Published:** 2022-07-25

**Authors:** Qian-Qian Deng, Mao Ye, Xiao-Bao Wu, Jia Song, Jun Wang, Li-Na Chen, Zhong-Yan Zhu, Jing Xie

**Affiliations:** a The Provincial Key Laboratory for Agricultural Pest Management of the Mountainous Region, Institute of Entomology, Guizhou University, Guiyang, China; b Scientific Observing and Experimental Station of Crop Pests in Guiyang, Ministry of Agriculture, Guiyang, China; c State Key Laboratory Breeding Base of Green Pesticide and Agricultural Bioengineering, Key Laboratory of Green Pesticide and Agricultural Bioengineering, Ministry of Education, Guizhou University, Guiyang, China

**Keywords:** Brown planthopper and leaffolder, specific anti-herbivore resistance, primed defense, signaling transduction pathways, ratoon rice, rice (*Oryza sativa*)

## Abstract

Herbivore-induced defense responses are often specific, whereas plants could induce distinct defense responses corresponding to infestation by different herbivorous insects. Brown plant hopper (BPH) *Nilaparvata lugens*, a phloem-feeding insect, and rice leaf folder (LF) *Cnaphalocrocis medinalis*, a chewing insect, are both specialist herbivores on rice. To characterize the distinct resistance primed by prior damage to these two specialist herbivores, we challenged rice plants with two herbivores during vegetative growth of parent plants and assessed plant resistance in subsequent ratoons. Here, we show that LF and BPH induce different suites of defense responses in parent rice plants, LF induced higher level of JA accumulation and *OsAOS, OsCOI1* transcripts, while BPH induced higher accumulation of SA and *OsPAL1* transcripts. Moreover, an apparent loss of LF resistance was observed in *OsAOS, OsCOI1* RNAi lines. Ratoon plants generated from parents receiving prior LF infestation exhibited higher jasmonic acid (JA) levels and elevated levels of transcripts of defense-related genes associated with JA signaling, while ratoon generated from parents receiving prior BPH infestation exhibited higher salicylic acid (SA) levels and elevated levels of transcripts of defense-related genes associated with SA signaling. Moreover, previous LF infestation obviously elevated ratoons resistance to LF, while previous infestation by BPH led to enhanced resistance in ratoons to BPH. Pre-priming of ratoons defense to LF was significantly reduced in *OsAOS* and *OsCOI1* RNAi plant, but silencing *OsAOS* and *OsCOI1* did not attenuate ratoons resistance to BPH. These results suggest that infestation of two specialist herbivores with different feeding styles in parent crop led to distinct defense responses in subsequent rations, and the acquired resistance to LF in ratoons is associated with priming of jasmonic acid-dependent defense responses.

## 1. Introduction

Herbivore-induced defense responses are often specific. While feeding, herbivores release a variety of cues present in their oral secretions, saliva and frass, which could specially mediates plant defense.^1^,^2^ Specificity of induced responses to herbivores in plants comprises two parts, the specificity of elicitation and the specificity of effect ^3–5^ specificity of elicitation is defined as distinct defense responses against different herbivores, for example, chewing and phloem-feeding insects cause different damage on plants and induce different suites of plant responses,^6,7^ chewing herbivores commonly induce a defense response mediated by the jasmonic acid (JA) pathway,^8^ whereas phloem feeders usually induce the salicylic acid (SA) pathway.^5,9,10^ The specificity of response and effect may be revealed through the expression of induced proteins, which leads to different effects on the growth of a herbivore. Antinutritive proteins, such as proteinase inhibitors (PIs), arginase (ARG), threonine deaminase (TD), or polyphenol oxidase (PPOs), are effective against specific proteases or in specific gut environments characteristic of the herbivore. Lepidopteran larvae often have alkaline midguts and rely on serine proteases; in contrast, coleopteran species normally have an acidic midgut and use cysteine and aspartic proteases as the major proteases for protein digestion.^3,11^ Chung found that the production of SerPI, ARG, TD and PPOs increased in plants damaged by tobacco hornworm (THW) compared to plants damaged by a Colorado potato beetle (CPB), indicating that tomato plants would produce distinct suites of antinutritive proteins in response to specific attacking herbivore.^1^

Plants can integrate environmental cues that predict upcoming herbivory to accelerate or enhance their induced defenses which is termed priming;^12–16^ numerous studies have shown that different defense responses are primed by different priming stimuli, upon a given biotic stress, to activate specific differences depending on the priming stimulus.^17–20^ In addition to pathogen challenge, primed responses can also be triggered by insect damage, insect oviposition and insect-induced volatiles. For instance, the egg-deposition of herbivorous insects on a host plant can prime plant defense against the feeding larvae, as has been shown for the wild tobacco *Nicotiana attenata*.^21,22^ Volatiles of herbivore-attacked plants can also prime herbivory-induced defense in adjacent plants.^23^ Evidence of the inherited responses to herbivory comes from research with wild radish (*Raphanus raphanistrun*), where insect-damaged plants produced more resistant seedlings than undamaged plants.^24^ In the case of *Arabidopsis*, prior-generation feeding by *Pieris rapae* (white cabbage butterfly) reduced caterpillar weight gain more than MeJA treatment and mechanical damage, in addition to *P. rapae*, only *S. exigua* showed reduced performance on plants that were exposed to *P. rapae* herbivory in the previous generation, these results confirmed the fact that there is species-specific variation in lepidopteran sensitivity to *Arabidopsis* defenses.^15^

Brown plant hopper (BPH) *Nilaparvata lugens*, a phloem-feeding insect, and rice leaf folder (LF) *Cnaphalocrocis medinalis*, a chewing insect, are both specialist herbivores on rice. In our previous study, we found that prior elicitation by LF infestation in rice parent plants enhances anti-herbivore resistance in subsequently generated ratoons through priming of JA-mediated defense.^25^ In the present investigation, we tested whether the prior elicitation by LF and BPH in parent rice plants would leave special imprints in the ratoons through epigenetic regulation of phytohormone-related genes, which induces distinct defense responses corresponding to distinct prior elicitation of different herbivores.

## 2. Materials and methods

### 2.1. Plant growth and generation of ratoons

The rice allene oxide synthase (*OsAOS*; GenBank accession no. AY062258) and CORONATINE INSENSITIVE1 (*OsCOI1*; GenBank accession no. DQ028826) genes were silenced by RNA interference (RNAi). The *OsAOS* and *OsCOI1* RNAi lines were generated as described by Ye *et al*.^26,27^

The rice genotypes used in this study were wild-type (WT) and *OsAOS* and *OsCOI1* RNAi lines. Pre-germinated seeds from the different lines were cultured in plastic buckets. Ten-day-old seedlings were transferred to plastic pots (diameter 30 cm, height 35 cm). The soil used for potting was obtained from the rice fields on the campus of Guizhou University in Guiyang, China. Plants were watered daily, and each pot was supplied with 20 mL of nutrient solution (urea, 1 g L^−1^) per week. All the plants were grown in a greenhouse (28°C, 14 h light, 10 h dark). After 25 days, the plants were infested by LF or BPH for 48 h. A second round of infestation were performed 15 d after the initial treatments, and then all parent plants were cut 2/3 above the ground for generation of ratoons as described in Ye *et al*.^25^

### 2.2. Insects rearing

Colonies of BPH and LF were originally obtained from the rice fields of the experimental farm of Guizhou University, Guiyang, China, and maintained on WT in a controlled climate chamber at 26°C, with a 12-h light phase and 80% relative humidity.

### 2.3. Insect infestation

*LF treatment*. Plants were individually infested using third-instar larva of LF that had been starved for 2 h and then placed on leaves at node 1, 2 and 3 (the youngest fully expanded leaf was defined as leaf 1), respectively, with one larva per leave.

*BPH treatment*. Plants were individually infested with 20 female adults of BPH that were contained in two parafilm bags (6 × 5 cm, with 60 small holes made by a needle, each bag containing ten females) fixed to upper and lower positions on the plant leaf sheaths. Two empty parafilm bags were attached to control plants (non-infested).

### 2.4. Quantitative real-time PCR

Relative expression levels of selected genes were determined by qRT-PCR by using total RNA samples isolated from the leaves or leaf sheath tissues of parent or ratoon plants. Total RNA was isolated using the RNAiso Plus reagent (Takara, Japan) according to manufacturer’s instructions. First-strand cDNA was synthesized from 1 µg of total RNA using MMLV-reverse transcription system (Promega, Madison, WI, USA) according to the manufacturer’s instructions. The qRT-PCR reactions were performed using the SYBR Premix EX TaqTM master mix (TaKaRa). The rice actin gene *OsActin* (GenBank accession no. X15865) was used as an internal standard to normalize cDNA concentrations. Reactions were performed using DNA Engine Opticon 2 Continuous Fluorescence Detection Syleaf sheaths (MJResearch Inc., Waltham, MA, USA). Gene specific primer sequences used for these experiments are listed in Table S1. The relative expression levels for genes of interest were calculated using the double-standard curves method. For qRT-PCR analysis, five independent biological samples were used. Reaction conditions for thermal cycling were as follows: 95°C for 1 min, 40 cycles of 95°C for 20 s and 58–60°C for 15 s.

### 2.5. JA and SA analyses

WT parent plants were randomly assigned to BPH or LF and control treatments. For plants infested by LF, node 3 leaves were harvested at 0, 3, 6, 12 and 24 h after LF infestation. For plants infested by BPH, leaf sheaths were harvested at 0, 3, 6, 12 and 24 h after BPH infestation. Leaves were collected and immediately flash-frozen in liquid nitrogen, and then stored at 80°C prior to analysis. Six plants were sampled at each time point from all treatments. Total JA and SA levels were analyzed by gas chromatography as previously described.^26^ Samples were extracted by mixture of acetone and citric acid (50 mmol L^−1^) (v/v = 7/3), and ethyl acetate. The supernatant was then dried by N_2_ and subsequently methylated with trimethylsilyldiazomethane. The volatilized compounds were collected using headspace-solid phase microextraction (HS-SPME) on Tenax adsorbents’ and eluted with n-hexane. Eluted samples were analyzed by using GC with hydrogen ion flame detector (FID). The temperature gradient was increased from 60°C (1 min) to 250°C in a rate of 15°C/min and held for 3 min at 250°C. The final chromatographic peaks of JA and SA in the samples were identical to the authentic compounds (Fig. S1). 25 ml 80 mg/ml JA and 125 ml 160 mg/ml SA were mixed, and after the step of extraction and methylation with trimethylsilyldiazomethane as samples, 100 ml n-hexane was used to elute the MeJA and MeSA collected in Tenax by HS-SPME, the mixed MeJA (20 mg/ml) and MeSA (200 mg/ml) were diluted into several concentration to be used as stands to quantify JA and SA levels of samples. In addition, mixed standard MeJA (18 mg/ml) and MeSA (40 mg/ml) (Sigma-Aldrich, St. Louis, MO, USA) were used to confirm the recovery rates of JA and SA. The method resulted in a high level of recovery, reproducibility, and linearity in the quantification of JA and SA (Fig. S2; Table S2).

### 2.6. Bioassays

LF performance measurement. WT and *OsAOS, OsCOI1* RNAi parent plants were individually infested using third instar LF larvae that had been weighed and starved to 2 h, and then placed on leaves. Larval mass was measured after 5 d of infestation, and the percent mass gain of larvae for each plant was calculated. Twenty plants of each genotype were used per treatment, and one larva per plant was inoculated. For LF performance measurement on ratoons, third instar LF larvae starved for 2 h were individually placed on leaves of WT and *OsAOS, OsCOI1* RNAi ratoons derived from un-infested, LF-infested and BPH-infested, larvae mass gain were measured and described as those on parent plants.

BPH performance measurement. The survival rates of BPH nymphs and honeydew secreted by female BPH on WT and *OsAOS, OsCOI1* RNAi parent or ratoon plants were determined. Newly emerging macropterous female BPH adults, starved for 2 h, were placed in to a small parafilm bag (6 × 5 cm), which was then fixed on the stems of plants, with each plant receiving three females. The amount of honeydew excreted by a female adult was weighed (to an accuracy of 0.1 mg) 24 h after the start of the experiment. The experiment was replicated 20 times.

The survival rates of BPH nymphs on were also determined. Pots with one plant were individually covered with a glass cylinder (diameter 13 cm, height 30 cm) into which 15 newly hatched BPH nymphs were released. The number of surviving BPH nymphs on each plant was recorded 5 d after insect infection. The experiment was replicated 10 times.

### 2.7. Data analysis

The SPSS 14.0 (SPSS) package for Windows was used for statistical analyses. Differential gene expression, JA and SA levels caused by LF or BPH at each time point as compared to control plants, respectively, were determined using Student’s *t*-tests. The LF and BPH performance of *OsAOS* and *OsCOI* RNAi parent plants as compared to WT plants, respectively, was determined using Student’s *t*-tests. Differential gene expression, JA and SA levels of LF- or BPH-infested and their respective non-infested WT control ratoons were determined by Tukey post-hoc test and one-way ANOVA at *P = .05*. LF and BPH performonce on *OsAOS* and *OsCOI* RNAi ratoons among genotypes were evaluated by factorial ANOVA with treatment differences among means tested at *P = .05* by using a Tukey post hoc test, and differentials among different treatments on each genotype were determined by Tukey post-hoc test one-way ANOVA at *P = .05.*

## 3. Results

### 3.1. OsAOS, OsCOI1, OsPAL1 *transcriptional responses and JA, SA accumulation in parent plants*

**Figure 1.**
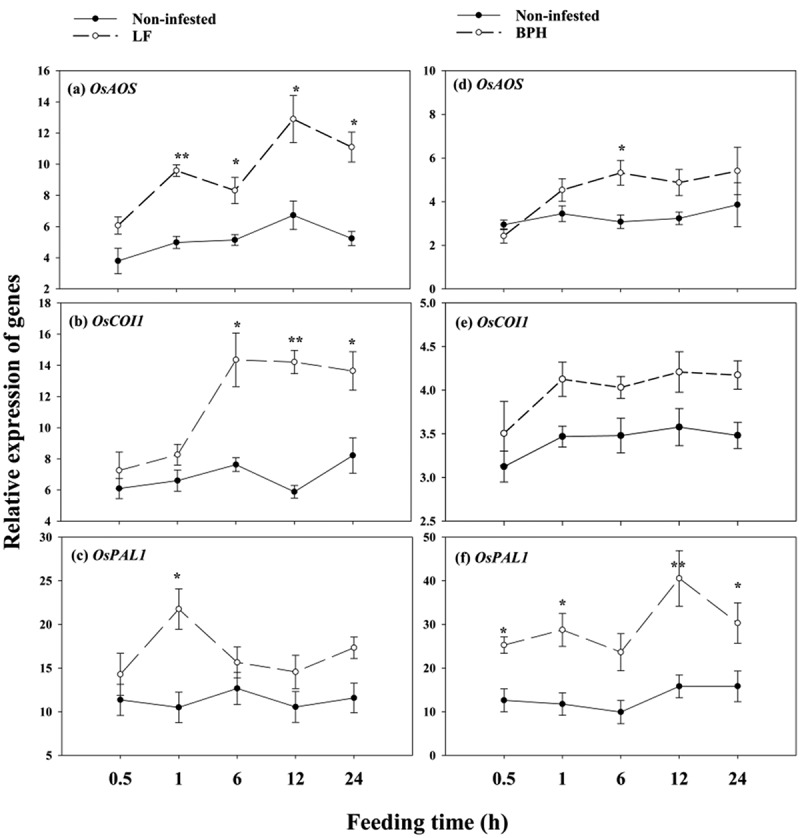
Expression of *OsAOS, OsCOI1* and *OsPAL1* in parent rice plants after LF (a, b, c) or BPH (d, e, f) infestation. Values are mean ± SE of six biological replicates. Asterisks indicate significant differences between infested and non-infested control plants (*P < .05; **P < .01, Student’s t-tests).

To determine transcript response of genes involved in JA and SA singling pathways to insect infestation in WT parent plants, we performed a time-course PCR analysis. Leaf tissue (or leaf sheath tissue) was harvested from individual plants at different time points after LF or BPH infestation. The results revealed that LF infestation led to increased *OsAOS* and *OsCOI1* transcript levels, BPH induces the expression of *OsAOS* at 6 h after infestation, but BPH infestation did not significantly change the transcript abundance of *OsCOI1* (Figure 1a,b,d,e). Compared to non-infested plants, *OsAOS* transcripts accumulated to 1.92, 1.61, 1.91 and 2.11-fold higher levels in response to LF infestation at 1, 6, 12 and 24 h, respectively (Figure 1a). *OsCOI1* transcripts accumulated to1.25, 1.88, 2.41 and 1.66-fold higher levels in response to LF infestation at 1, 6, 12 and 24 h, respectively (Figure 1b). Actually, LF can also induce the expression of *PAL1* at 1 h (Figure 1c). *OsPAL1* transcripts were significantly induced upon BPH attack. Its transcripts were 2.44, 2.38, 2.56 and 1.91-fold higher at 1, 6, 12 and 24 h, respectively (Figure 1f).

**Figure 2.**
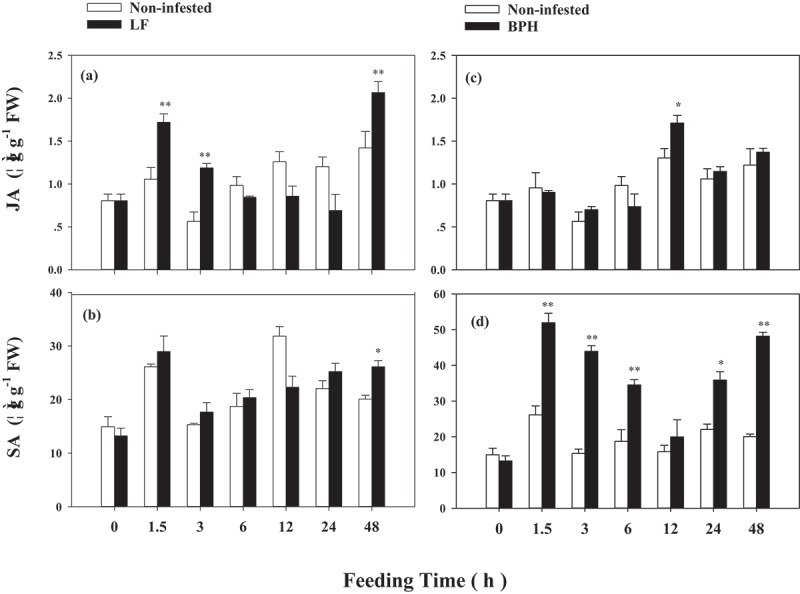
Herbivore induced jasmonic acid (JA) and salicylic acid (SA) accumulation in parent rice plants in response to LF (a, b) or BPH (c, d) infestation. Values are mean ± SE of six biological replicates. Asterisks indicate significant differences between treatments and non-infested controls (*P < .05; **P < .01, Student’s t-tests).

To determine whether the JA and SA biosynthesis were deferentially induced by LF and BPH infestation, we investigated JA and SA levels. Compared to non-infested plants, LF damage induced 0.63, 1.10 and 0.45-fold higher JA levels at 1.5, 3 and 48 h, respectively (Figure 2a); however, LF damage just induce 0.30-fold higher SA accumulation at 48 h (Figure 2b). BPH infestation led to a higher accumulation of SA, SA levels were 0.98, 1.86, 0.85, 0.63 and 1.40-fold higher than those of non-infested plants at 1.5, 3, 6, 24 and 48 h, respectively (Figure 2d), and higher JA levels were only observed at 12 h after infestation(Figure 2c).

### 3.2. *Silencing* OsAOS *and* OsCOI1 *reduces resistance to LF but not to BPH in parent rice plants*

**Figure 3.**
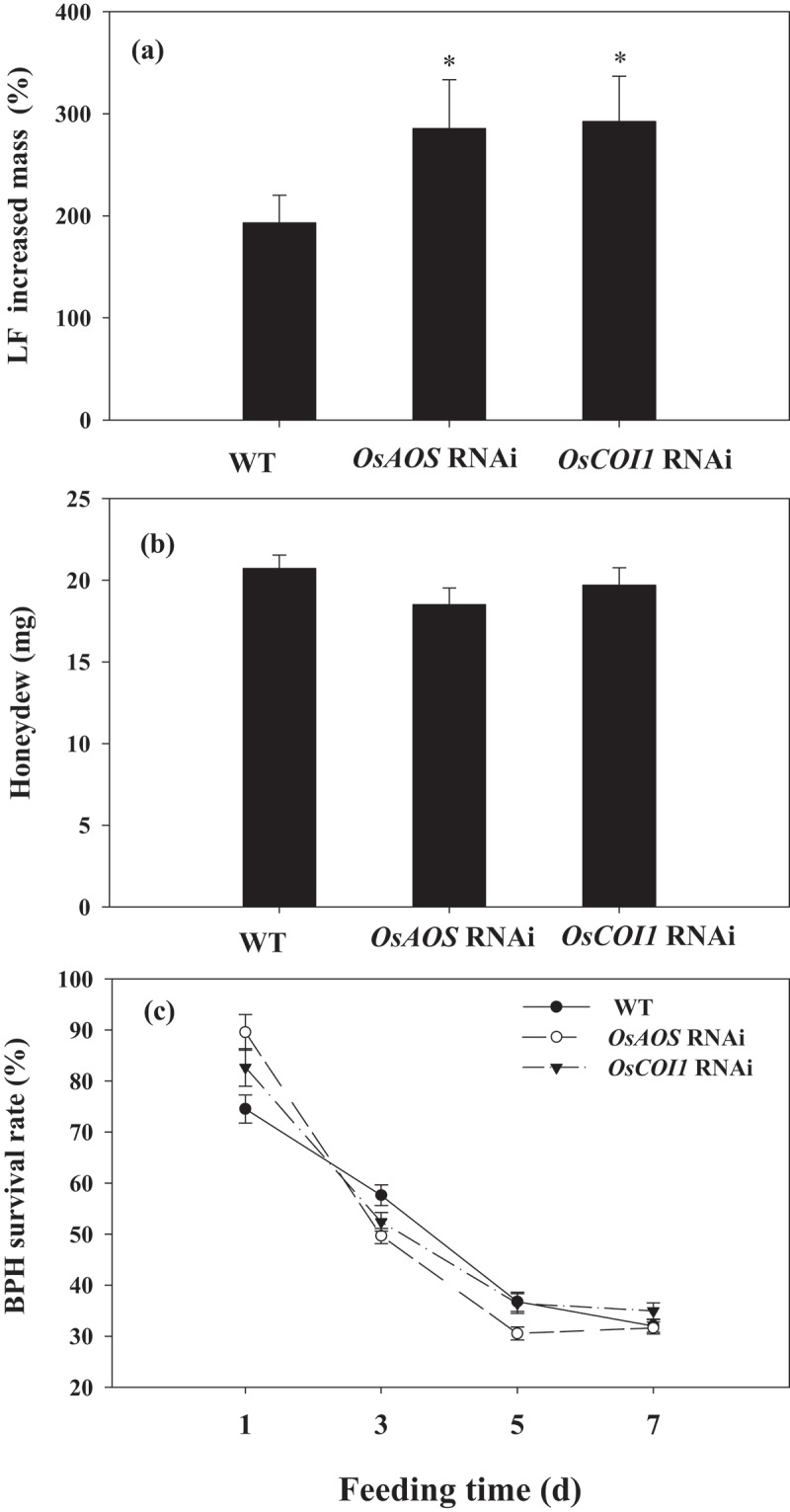
*OsAOS* and *OsCOI1* positively regulates rice resistance to LF (a) but negatively to BPH (b, c). Values are mean ± SE. (a) Mean larval mass (n = 60) (b) Mean amount of honeydew per day (n = 15). (c) Mean survival rate (n = 20) . Asterisks indicate significant differences in *OsAOS* and *OsCOI1* RNAi lines compared with WT plants (*P < .05; **P < .01, Student’s t-tests).

LF larvae fed on *OsAOS* and *OsCOI1* RNAi rice plants increased in mass 285.37% and 292.32% by 5 d after infestation, whereas larvae fed on WT plants increased in mass by 193.13% (Figure 3a). We also assessed the performance of BPH on *OsAOS* and *OsCOI1* RNAi rice plants.The results revealed that the amounts of honeydew secreted per day by a BPH female adult on *OsAOS* and *OsCOI1* RNAi did not differ from those fed on WT plants (Figure 3b), and the survival rates of BPH nymphs fed on *OsAOS* and *OsCOI1* RNAi lines also showed no significant difference with those fed on WT plants (Figure 3c). The obvious differences in LF weight gain between those feeding on WT and RNAi rice plants demonstrate the important role of *OsAOS* and *OsCOI1* in rice resistance against LF. While the results show that BPH shows no difference between WT and RNAi rice plants suggesting that the reduced expression of *OsAOS* or *OsCOI1* does not negatively affect rice resistance against BPH.

### 3.3. Transcripts of JA and SA-responsive genes in WT rice ratoons to LF or BPH exposure

**Figure 4.**
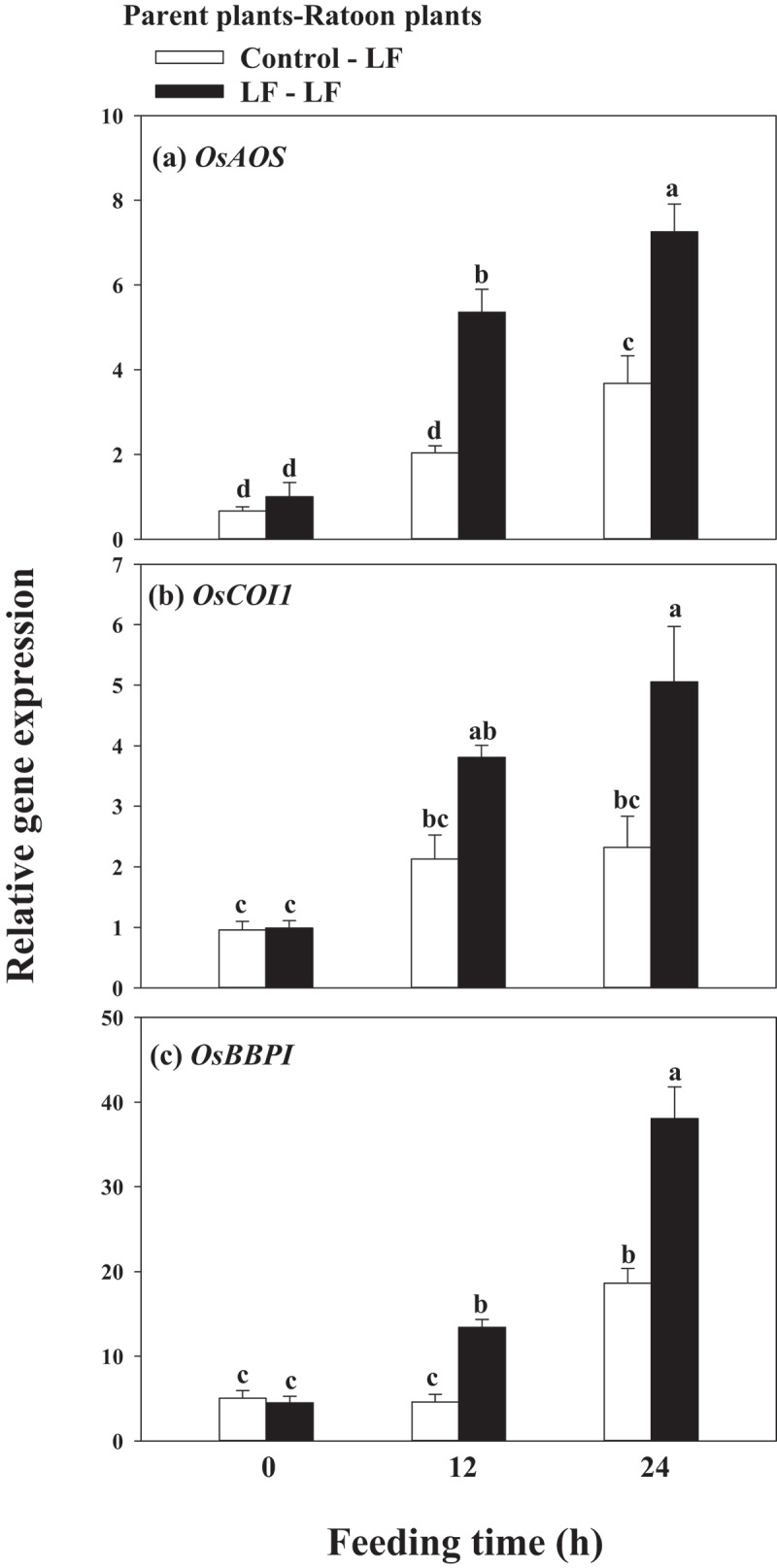
Expression of *OsAOS* (a), *OsCOI1* (b) and *OsBBPI* (c) in ratoon rice plants in response to LF infestation. Values are mean ± SE of six biological replicates. Letters above bars indicate significant difference among treatments (*P < .05* according to Tukey’s multiple range test one-way ANOVA).

To determine whether a special infestation of LF and BPH during the vegetative growth phase of parent plants could activate distinct defense in the ratoons, we challenged WT rice plants with LF or BPH, transcripts of JA and SA-responsive genes were tested in subsequent ratoons in response to LF or BPH infestation. Compared with control ratoon plants without prior LF infestation, *OsAOS* transcript levels in the ratoon plants with prior LF infestation increased by 1.62-fold and 0.97-fold after 12 and 24 h of LF exposure, respectively (Figure 4a), and *OsCOI1* increased by 0.79-fold and 1.18-fold after 12 and 24 h of LF exposure, respectively (Figure 4b). Protease inhibitors (PIs) have been implicated in plant defense against lepidopteran herbivorous insects via interfering with their digestive process,^28^ we found that *OsBBPI* transcript levels in ratoons derived from parent plants with prior LF infestation increased by 1.91-fold and 1.04-fold after 12 h and 24 h of LF exposure, respectively, relative to ratoons without prior LF infestation (Figure 4c).

**Figure 5.**
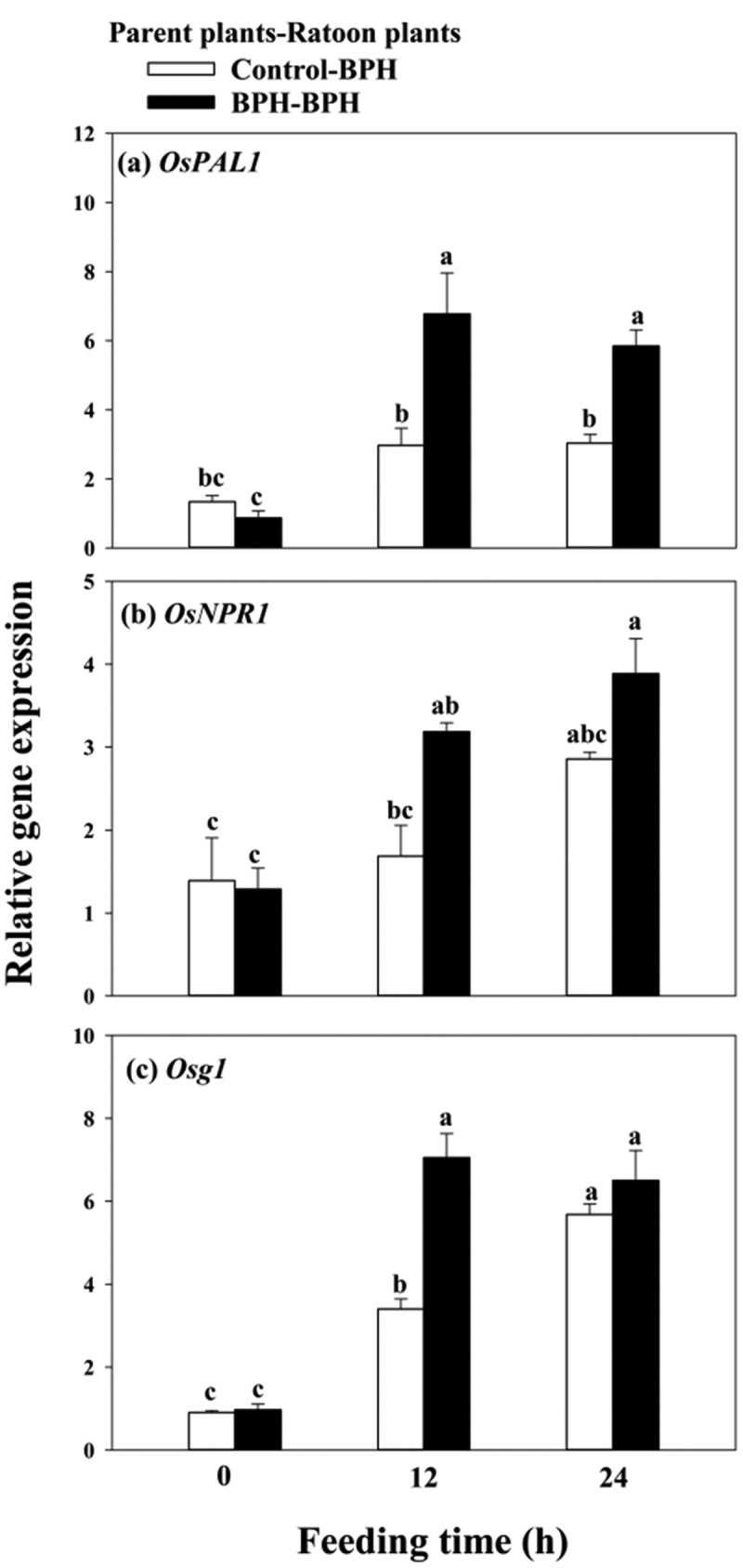
Expression of *OsPAL1* (a), *OsNPR1*(b) and *OsOsg1*(c) in ratoon rice plants in response to BPH infestation. Values are mean ± SE of six biological replicates. Letters above bars indicate significant difference among treatments (*P < .05* according to Tukey’s multiple range test one-way ANOVA).

Additionally, to determine whether prior BPH infestation occurred in parent plants primed rice resistance in the corresponding ratoons to BPH, ratoon plants were all infested by BPH. In ratoon plants with prior BPH infestation, OsPAL and OsNPR1 transcript levels increased by 1.28-fold and 0.89-fold after 12 h of BPH exposure, respectively (Figure 5a,b), relative to control ratoon plants without prior BPH infestation. Callose deposition is a dynamic stress response of the plants to herbivorous infestation, especially to piercing sucking insects. Osg1 is found to be expressed throughout the plant and highly expressed in leaf sheaths,^29^ and could be strongly induced by BPH.^30^ Here, we found that transcript levels of Osg1 in ratoons derived from parent plants with prior BPH infestation increased by 1.07-fold after 12 h of BPH exposure, relative to ratoon plants without prior BPH infestation (Figure 5c)

### 3.4. JA and SA accumulation in WT rice ratoons to LF or BPH exposure

**Figure 6.**
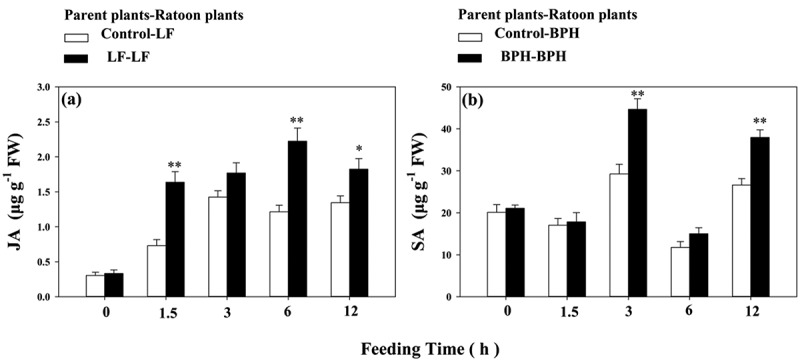
Jasmonic acid (JA) and salicylic acid (SA) accumulation in ratoon rice plants in response to LF (a) or BPH (b) infestation.Values are mean ± SE of six biological replicates. Asterisks indicate significant differences between infested and non-infested controls (*P < .05; **P < .01, Student’s t-tests).

LF infestation led to increased JA accumulation levels at all time points examined in ratoons derived from parents not exposed to LF (Figure 6a). Significantly higher JA levels were observed in ratoons derived from LF-exposed parents (Figure 6a). For example, in ratoon plants with prior LF infestation, at 1.5 h following LF exposure, JA accumulation levels were 2.24-fold higher than those observed in ratoons without prior LF infestation. Similar to the observed increases in *OsPAL* and *OsNPR1* transcript levels, BPH infestation led to increased SA accumulation levels at all time points examined in ratoons derived from parents not exposed to BPH (Figure 6b). For example, in ratoon plants with prior BPH infestation, at 3 h following BPH exposure, SA accumulation levels were 1.53-fold higher than those observed in ratoons without prior BPH infestation (Figure 6b).

### 3.5. Special induction in parent plants activate specific differences priming in the ratoons

**Figure 7.**
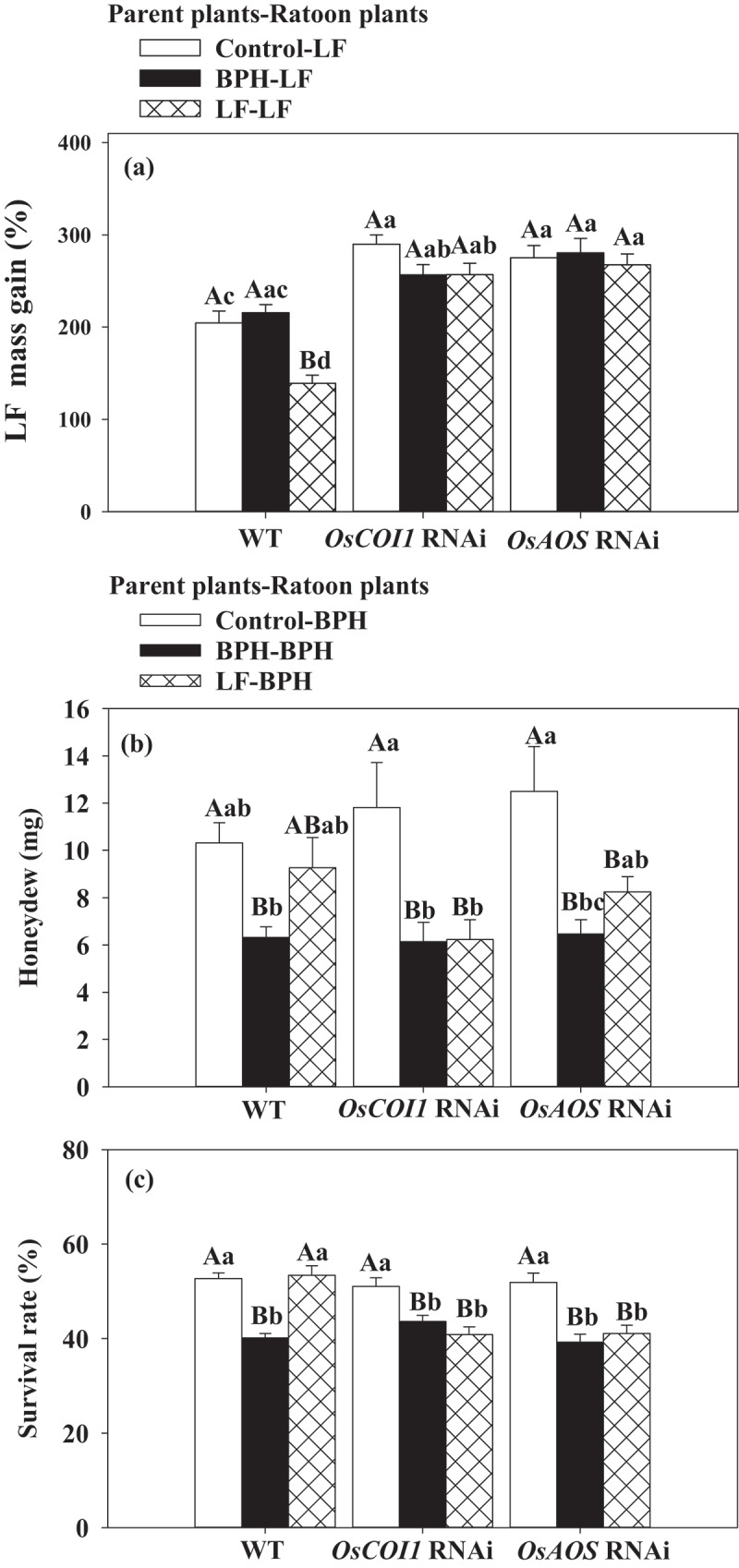
Performance of LF and BPH on WT and *OsAOS, OsCOI1*-deficient ratoons generated from LF (a) or BPH (b, c) infested parents. Values are mean ± SE. (a)Mass gain of LF larvae (n = 60). (b) Mean amount of honeydew per day (n = 15) and (c) mean survival rate (n = 20). Capital letters above bars indicate significant difference among treatments in each genotype (*P < .05* according to Tukey’s multiple range test one-way ANOVA), lowercase letters indicate significant difference among treatments among genotypes (*P < .05* according to Tukey’s multiple range test factorial one-way ANOVA).

To determine whether special infestation of LF and BPH during the vegetative growth phase of parent plants could activate specific differences priming in the ratoons, rice seedlings of three genotypes (WT, *OsAOS*-deficient and *OsCOI1*-deficient RNAi lines) were infested by LF or BPH. The results showed that LF infestation of WT parent plants significantly increased rice ratoons resistance to LF but not to BPH, while BPH infestation of WT parent plants significantly increased ratoons’ resistance to BPH but not to LF. LF larvae fed on ratoons derived from un-infested and BPH-infested parents increased in mass 204.48% and 215.47% 5 d after infestation, respectively, whereas larvae fed on ratoons derived from LF-infested parents increased in mass by only 138.92% (Figure 7a). There were no significant differences in honeydew and survival rate of BPH between those fed on ratoons derived from un-infested or LF-infested WT parents. Silencing *OsCOI1* and *OsAOS* expression significantly reduced the resistance of rice ratoons to LF infestation, irrespective of the parent plant infestation treatment performed (Figure 7a). LF larvae fed on *OsCOI1* and *OsAOS* RNAi ratoons derived from control parent plants without prior LF infestation increased in mass by 272.40% and 286.63% 5 d after infestation, respectively; however, prior LF and BPH infestations did not significantly affect ratoon resistance levels against LF on *OsCOI1* and *OsAOS* RNAi lines, suggesting that the observed increases in ratoon LF resistance occur in a JA-dependent manner.

BPH infestation to WT parent plants significantly increased ratoons’ resistance to BPH. The amounts of honeydew secreted per day by a BPH female adult fed on ratoons derived from BPH-treated parents was 38.82% lower than those fed on rice ratoons from un-infested parents (Figure 7b), also, the survival rate of BPH nymphs was 21.64% lower (Figure 7c). However, LF infestation of WT parent plants did not significantly affect ratoon BPH resistance levels, the honeydew and the survival rate of BPH fed on ratoons with prior LF infestation showed no significant difference form those fed on ratoons not prior infested by LF (Figure 7b,c). Silencing *OsAOS* and *OsCOI1* did not reduce resistance to BPH on ratoons derived from control parents without infestation, but honeydew secreted by female adult fed on *OsAOS*-deficient and *OsCOI1*-deficient ratoons derived from BPH-infested parent was 48.23% and 48.06% lower than those fed on ratoons from uninfested parent, respectively (Figure 7b), also, the survival rate of BPH nymphs was 24.35% and 14.53% lower (Figure 7c), suggesting that the resistance of ratoons to BPH primed by prior BPH infestation is not regulated by JA. However, *OsAOS* and *OsCOI1* RNAi ratoons derived from LF-infested patents showed increased resistance to BPH, honeydew of BPH female adult reduced 37. 02% and 47.24% (Figure 7b), and survival rate of BPH reduced 20.81% and 19.98%, respectively (Figure 7c).

## 4. Discussion

To offset their sessile life, plants have evolved diverse strategies to survive and adapt to insect herbivory.^31^ Upon attack, plants initiate defense responses aided by the recognition of herbivore-specific molecular patterns,^32–34^ followed by the activation of a complex regulatory network involving JA, SA and ET signaling, the expression of defense-related genes, and the production of defense compounds.^35^ Previous defense induction can cause plants to be primed for a more robust or rapid defense response upon subsequent attack,^14^ while different priming stimuli activate specific differences depending on the priming stimulus,^19,36^ here, we examined whether the prior damage by two specialist herbivores (LF and BPH) in parent rice plants would leave special imprints in the plant’s phytohormonal regulation in subsequent ratoon rice. We found that the two specialist herbivores on rice, LF and BPH induce different suites of defense responses in parent rice plants, leading to specific resistance to the two herbivores in subsequently generated ratoons. Additionally, using JA-deficient *OsAOS* and *OsCOI1* RNAi lines, we demonstrate that the perception and regulation of JA is required for increased resistance in ratoon rice plants to LF, but not to BPH.

Our results showed that LF induced higher *OsAOS, OsCOI1* transcripts level and JA accumulation (Figure 1a,b; Figure 2a), while BPH induced higher *OsPAL1* transcripts level and SA accumulation in WT parent plants (Figure 1f; Figure 2d), also, obvious increased mass of LF were found on *OsAOS* and *OsCOI1* RNAi lines (Figure 1a). The differential activation of signaling pathways elicited by LF and BPH suggests that JA and SA signal pathways play different roles in rice resistance against these two specialist pests; these results are in line with the prior reports that JA-mediated defense signaling pathway has a central role in chewing herbivore attack, whereas SA signaling pathway is more specific to piercing sucking herbivores.^33,34^

Accumulated studies reported that once anti-herbivore defenses have been triggered either by herbivore encounters or elicitor treatment, defense-associated pathways may maintain a primed state persisting throughout the life cycle of the plant, including expression within organs and tissues formed long after the initial exposure.^37–40^ In our study, we found that prior LF infestation in parent rice plants leads to higher transcripts of *OsAOS, OsCOI1* and JA mediated-*OsBBPI* in ratoons in response to LF attack (Figure 4a,b,c), whereas prior BPH infestation in parent rice plants leads to higher transcripts of *OsPAL1, OsNPR1* (Figure 5a,b) and SA mediated-*Osg1*(Figure 5c) in ratoons to BPH attack, suggesting that the differential activation of JA and SA signaling pathways in response to different elicits in parent plants allows species-specific responses to different herbivores.

Studies have shown that the main reasons for the next generation of *Arabidopsis* to acquire defensive capabilities are the accumulation of JA, the expression of JA-related insect defense genes, and the production of glucosinolates in leaves.^15^ Here in this study, we observed priming for faster and higher JA induction in response to LF attack on ratoon plants derived from LF infested parent plants (Figure 6a), and higher SA induction in response to BPH attack on ratoon plants derived from BPH infested parent plants (Figure 6b). Furthermore, an apparent increase in resistance to LF was observed in WT ratoons derived from LF-infested parents (Figure 7a), but WT ratoons resistance to BPH was not significantly affected by prior LF infestation (Figure 7b,c). Silencing expression of *OsAOS* and *OsCOI1* attenuated ratoons resistance to LF but had no significant affect on the resistance of ratoons to BPH (Figure 7a), but the decreased expression of *OsAOS* and *OsCOI1* did not affect ratoons’ resistance against BPH (Figure 7b,c); likewise, prior BPH infestation enhanced ratoons’ defenses to BPH but not to LF (Figure 7b,c), these suggest that the mechanism underlying the specific priming on ratoons is mediated by corresponding signaling pathways elicited by two insects in parent plants.

Accumulating evidence demonstrates cross-talk between different phytohormone signaling pathways. JA- and SA-dependent signaling pathways can be antagonistic when directly activated.^41^ Zhou *et al*. found that silencing of the key JA gene *OsHI-LOX* reduced rice resistance against SSB (*Chilo suppressalis*) and LF, while the content of SA was relatively increased, which enhanced the resistance of rice to BPH.^42^ Meanwhile, Xu *et* *al.* (2021) reported that JA mutant significantly affected rice resistance to BPH, and SA-depleted rice plants did not affect resistance to BPH.^43^ Recently, Bai *et* *al.* (2022) reported that JA is a major component in tobacco defending against leafhopper, a sucking insect.^44^ Actually, BPH can induce the expression of *OsAOS* at 6 h after infestation (Figure 1d), and LF can also induce the expression of *OsPAL1* at 1 h (Figure 1c), while we did not find obviously higher SA accumulation in parent plants infested by LF (Figure 2b), in addition, silencing of *OsAOS* and *OsCOI1* did not enhance parents’ (Figure 3b,c) and ratoons’ resistance to BPH (Figure 7b,c); thus, our data indicates a complex interaction between JA and SA signaling during infestation of these two insects with different feeding style, further work using SA deficient mutants will be needed to determine whether the priming resistance against BPH requires SA perception in subsequently generated ratoons.

## Supplementary Material

Supplemental Material
