# Supplementary material for: Damage of brown planthopper (BPH) Nilaparvata lugens and rice leaf folder (LF) Cnaphalocrocis medinalis in parent plants lead to distinct resistance in ratoon rice
Source: Plant Signal Behav. 2022 Jul 25;17(1):2096790. doi: 10.1080/15592324.2022.2096790 (PMC9318313; doi:10.1080/15592324.2022.2096790)
Supplement: Supplemental Material [file KPSB_A_2096790_SM4580.doc]

**Figure S1. Profiles of GC chromatography of authentic MeJA, MeSA and JA derived MeJA and SA derived MeSA in rice leaves.**

**Figure S2. Recovery rates of jasmonic and salicylic acids in the GC analysis**

**Table S1. Specific Primer for Real-Time PCR**

| **Gene** | **TIGR ID** | **Sequence of Primer** |
| --- | --- | --- |
| *OsAOS* | Os03g12500 | 5‘-atgtatcggatcaatctgcccta -3  5’-gaaggacgacgacaaagaagaaa -3’ |
| *OsCOI1* | Os01g63420 | 5‘-ttgccgtgaattggagtacatag-3'  5’-gtcaagtagcacaagccgaaag-3' |
| *OsPAL1* | Os02g41630 | 5‘-ttggcgatggcgagac-3'  5’-ctggatggtgcttgagcttgg-3' |
| *OsNPR1* | Os01g09800 | F:5‘-3’tggacaggttatcaccattggt  R:5’-3’ccgcagcttccattcctatg |
| *OsBBPI* | Os01g71930 | 5’- gctcatctgcgaggacatct -3’  5’- ttcctcatggtccacacaag -3’ |
| *Osg1* | Os05g27730 | 5'-gaccaatgtgcaagcctttt-3'  5'-gtccctttgctcacatggtt-3' |
| *OsACT* | Os03g50885 | 5'-tggacaggttatcaccattggt-3'  5'-ccgcagcttccattcctatg-3' |

**Table S2 Retention time, linear regression equation and limit of detection of JA and SA detected by GC-FID**

| Compound | Retention time | Linear regression | Linearity range | Coefficient | Detection limit |
| --- | --- | --- | --- | --- | --- |
|  | (min) |  | (ng μl^-1^) |  | (ng μl^-1^) |
| Salicilic acid | 7.271 | Area=30.1200121×Amt-25.753772 | 0.5-40.0 | 0.99921 | 0.100030 |
| Jasmonic acid | 11.003 | Area=37.3437531×Amt-5.0685045 | 0.225-18.000 | 0.99986 | 0.080680 |
